# A novel 3D histotypic cartilage construct engineered by supercritical carbon dioxide decellularized porcine nasal cartilage graft and chondrocytes exhibited chondrogenic capability *in vitro*

**DOI:** 10.7150/ijms.56342

**Published:** 2021-03-25

**Authors:** Su-Shin Lee, Yi-Chia Wu, Shu-Hung Huang, Ying-Che Chen, Periasamy Srinivasan, Dar-Jen Hsieh, Yi-Chun Yeh, Yi-Ping Lai, Yun-Nan Lin

**Affiliations:** 1 Division of Plastic Surgery, Department of Surgery, Kaohsiung Medical University Hospital, Kaohsiung City, Taiwan.; 2 Department of Surgery, Faculty of Medicine, College of Medicine, Kaohsiung Medical University, Kaohsiung City, Taiwan.; 3 Regenerative medicine and cell therapy research centre, Kaohsiung Medical University, Kaohsiung, Taiwan.; 4 Department of Surgery, Kaohsiung Municipal Siaogang Hospital, Kaohsiung, Taiwan.; 5 Center of Research and Development, ACRO Biomedical Co., Ltd. Kaohsiung, Taiwan.

**Keywords:** supercritical carbon dioxide, 3D histotypic cartilage, decellularized porcine nasal cartilage graft (dPNCG), chondrocytes, adipose-derived stem cells

## Abstract

Augmentative and reconstructive rhinoplasty surgical procedures use autologous tissue grafts or synthetic grafts to repair the nasal defect and aesthetic reconstruction. Donor site trauma and morbidity are common in autologous grafts. The desperate need for the production of grafted 3D cartilage tissues as rhinoplasty grafts without the adverse effect is the need of the hour. In the present study, we developed a bioactive 3D histotypic construct engineered with the various ratio of adipose-derived stem cells (ADSC) and chondrocytes together with decellularized porcine nasal cartilage graft (dPNCG). We decellularized porcine nasal cartilage using supercritical carbon dioxide (SCCO2) extraction technology. dPNCG was characterized by H&E, DAPI, alcian blue staining, scanning electron microscopy and residual DNA content, which demonstrated complete decellularization. 3D histotypic constructs were engineered using dPNCG, rat ADSC and chondrocytes with different percentage of cells and cultured for 21 days. dPNCG together with 100% chondrocytes produced a solid mass of 3D histotypic cartilage with significant production of glycosaminoglycans. H&E and alcian blue staining showed an intact mass, with cartilage granules bound to one another by extracellular matrix and proteoglycan, to form a 3D structure. Besides, the expression of chondrogenic markers, type II collagen, aggrecan and SOX-9 were elevated indicating chondrocytes cultured on dPNCG substrate facilitates the synthesis of type II collagen along with extracellular matrix to produce 3D histotypic cartilage. To conclude, dPNCG is an excellent substrate scaffold that might offer a suitable environment for chondrocytes to produce 3D histotypic cartilage. This engineered 3D construct might serve as a promising future candidate for cartilage tissue engineering in rhinoplasty.

## Introduction

Rhinoplasty is the procedure that alters and restructures the defected nose for the form and function caused by trauma, therapeutic operations including cancer, dermatological disease, congenital malformations and plastic surgeries, the most prevalent in the cosmetic field. Nasal defects in patients cause overwhelming facial deformity leading to physical and psychological problems disturbing their quality of life, interpersonal relationships and workability. The main goal of rhinoplasty is to produce a clear nasal airway passage for the patient, accomplish wound healing, an unobtrusive nose to progress in their self-confidence and quality of life. Augmentative rhinoplasty is one of the most famous cosmetic surgery procedures in Asia 1, 2.

Dorsal augmentative rhinoplasty is the most common protocol, in which a nasal implant is implanted into a pocket created in a subperiosteal region of the nose. Majorly, there are two types of nasal implants employed for this surgical procedure, the autologous cartilage graft and the synthetic nasal implant 2. The autologous cartilage graft is derived from the patients' own tissue that includes the auricular or costal cartilage. Autologous cartilage graft causes significant wound pain in addition to donor site morbidity, long surgery time, inadequate reconstructive possibilities due to the minimal availability of the cartilage. Synthetic nasal implant possesses limitations such as integration with the host tissue, high levels of infection and extrusion 1. Hence, there is an unmet clinical need to produce appropriate material for nasal reconstruction to offer improved results for patients 3. Therefore, the focus has been shifted towards the use of decellularized xenogenic cartilage scaffolds in recent years, because of their large availability and improved decellularization process without damaging the scaffold with little or no adverse effects 1-3.

In recent years, natural extracellular matrix derived scaffolds are gaining popularity owing to their natural structural composition and native microenvironment 4. Decellularization technology is used to reduce the antigenicity of xenogeneic tissues and retain all the important compositions of the extracellular matrix 5. Porcine septal cartilage is produced as a decellularized extracellular cartilage matrix that is appropriate for cartilage tissue engineering with human chondrocytes. Decellularized septal cartilage was produced using strong denaturant and base, guanidine hydrochloride 6. However, not all the decellularization technology was found to be efficient and good for the production of natural scaffolds, that can be used for tissue engineering.

The challenge in cartilage decellularization is its dense structure with separate lacunae. Essentially, cartilage decellularization is achieved by diffusion of detergents into the lacunas to break the chondrocyte cell wall and subsequent washing to remove the cell debris and DNA. The decellularization process of cartilage includes a blend of physical, chemical, and enzymatic stages. The detergents and chemicals used in the decellularization process destroy the unique scaffold architecture of the cartilage 7. These disadvantages can be excluded by employing supercritical carbon dioxide (scCO_2_) technology for nasal cartilage decellularization to produce decellularized nasal cartilage scaffold.

Supercritical carbon dioxide (scCO_2_) extraction technology has demonstrated to be an outstanding alternate process for decellularization; because it is environmentally friendly, non-toxic, higher yield, cost-effective and efficient in eliminating toxic chemicals and retaining extra-cellular matrix (ECM) components. ScCO_2_ technology is advantageous, with no solvent residue, off-odours, and complete solubilization and removal of lipids, while maintaining the protein scaffold structurally. Besides, the scCO_2_ method destroys and eradicates pathogens 5, 8. This study aimed to develop a non-toxic, biocompatible decellularized nasal cartilage scaffold matrix that could be used in tissue-engineered 3D histotypic construct for rhinoplasty.

## Materials and methods

### Preparation of decellularized porcine cartilage

The decellularized porcine nasal cartilage graft (dPNCG) was produced by ACRO Biomedical Co. (Taiwan). The source of the porcine tissue was procured from Tissue Source, LLC (Lafayette, Indiana 47909). The porcine nasal cartilage was surgically removed using a scalpel and washed using sterilized water. The porcine nasal cartilage was decellularized as described previously 5, 8-10. The decellularized porcine nasal cartilage was freeze milled into powder form for the current study.

### Hematoxylin and eosin staining

dPNCG and normal nasal cartilage were subjected to routine 4% buffered formaldehyde fixation and followed by paraffin embedding. Hematoxylin and eosin (H&E) were performed using the sections of 5 µm thickness of the paraffin block for evaluation of complete decellularization. The slides were photographed using a microscope (Olympus bx53) for further analysis.

### DAPI staining

dPNCG and normal nasal cartilage sections were made as mentioned in the aforementioned section. Following the manufacturer's instructions, the paraffin sections were stained with 4,6-Diamidino-2-phenylindole (DAPI). The slides were photographed using a fluorescence microscope in the darkroom for further analysis.

### Alcian blue stains for glycosaminoglycans (GAGs)

dPNCG and normal nasal cartilage sections were made as in the aforementioned section. Alcian blue solution (pH 2.5) was used as a primary stain and counterstained with the nuclear fast red for evaluation of GAGs. The stained sections were photographed using a microscope (Olympus bx53) for further analysis.

### Scanning electron microscopy (SEM)

dPNCG and normal nasal cartilage were prepared for SEM following standard procedure. Microstructural features of dPNCG and normal nasal cartilage were scanned using a field emission SEM (Hitachi S-3400 N). SEM images of the surface of dPNCG and normal nasal cartilage samples at different magnifications were recorded 5.

### DNA quantification

The genomic DNA was extracted from dPNCG and normal nasal cartilage using a commercial kit (NautiaZ Tissue DNA Mini Kit, Nautiagene, Taipei, Taiwan). Using a microplate reader the extracted DNA was quantified by measuring at 260 nm.

### Protein content analysis by sodium dodecyl sulphate-polyacrylamide gel electrophoresis (SDS-PAGE)

The protein content of dPNCG and native nasal cartilage was analysed by a standard SDS-PAGE (8%). We used type I collagen and molecular weight marker for standard comparison. The dPNCG sample was prepared by dissolving 100 mg of decellularized cartilage in 0.01N HCl with 10 mg pepsin at 4°C overnight.

### Rat adipose-derived stem cells (ADSC) isolation and expansion

The ADSCs were prepared as described in Sheen et al. and Chang et al. 11, 12 Briefly, adipose tissues were excised from the rat bilateral groin area. The samples were digested at 37 °C in 0.1% collagenase (Sigma-Aldrich, St. Louis, MO, USA) with vigorous shaking for 90 min. The digested tissues were mixed with Dulbecco's modified Eagle's medium (DMEM) supplemented with 10% fetal bovine serum (FBS), and centrifuged at 1500rpm for 5 min. The cells were suspended in DMEM containing 10% FBS and filtered through a 100 μm cell strainer. Then cells were plated and cultured in DMEM containing 10% FBS at 37 °C in a humidified incubator with 5% CO_2_. The cell culture medium for putative ADSCs, referred to as chondrogenic differentiation medium, was a modified DMEM contained 10 ng/ml TGF-β1 and 50 µg/ml L-ascorbic acid 2-phosphate, and the medium was changed every other day until confluence. The fourth-passage ADSCs were used in this study.

### Characterization of ADSCs by flow cytometry

ADSCs were characterized by flow cytometry at passage 4. Briefly, ADSCs were harvested and washed twice with phosphate-buffered saline (PBS). Then, the cells were incubated for 30 min in PBS containing anti-CD45-FITC (cat. #554878, BD Biosciences USA), Mouse Anti-Rat CD31 (cat. # 25-0310-80, BD Bioscience USA), Anti-Rat CD11b (cat. #562105, BD Biosciences, USA), Mouse Anti-Rat CD90 (cat. #551401, BD Biosciences USA), CD105 (cat. # 05-1424, Millipore USA). The stained cells were then subjected to flow cytometry analysis.

### Rat chondrocyte isolation and expansion

The rat cartilage was obtained using a sterile sharp scalpel blade from rib cartilage of Lewis rat, finely minced and placed in an antimicrobial solution (20% Antibiotic-Antimycotic in PBS) for 4 hrs. Chondrocytes were isolated by the enzymatic digestion of the cartilage tissue extracellular matrix using purified collagenase solution (SIGMA C2674 1G) at 37 °C overnight.

Chondrocytes were filtered via a 100 μm cell strainer from the digested cartilage tissue. The filtered chondrocytes were washed with 2% PSA-PBS and centrifuged at 1500 rpm, 5 min. The supernatant was removed and the pellet was rinsed with culture medium (DMEM supplemented with 10% FBS, 1% L-glutamine) twice and centrifuged at 1500 rpm for 5 min. Chondrocytes suspended in the medium were counted. Cells were seeded in a 10 cm petri dish or 75T flask at a density of 15,000 cells/cm^2^ with culture medium and incubated in 5% CO_2_ at 37°C. Cells between passages 2-3 were used for the analysis. Cell debris and non-adherent cells were removed after 24 h and the culture medium was changed every 3 days. The culture plates were maintained at 37 °C with primary chondrocyte cultures as a high-density monolayer before encapsulation in the 3D composite.

### Characterization of chondrocytes by flow cytometry

Cells were plated at a density of 200 cells/cm^2^ on culture dishes and harvested at about 50% confluence. 10^5^-10^6^ cells were incubated with FITC- or PE-conjugated anti- CD45 and anti- CD44 for 30 min at 4 °C in PBS. The primary and secondary antibodies concentrations used were as follows: rat anti- CD45 (cat. #554878, BD Biosciences USA), and rat anti- CD44 dilution 1:200 (cat. #550974, BD Biosciences, USA) Isotype-matched irrelevant polyclonal antibodies were used as negative controls. For cell surface staining, cells were incubated in darkness for 30 min at 4 °C in PBS supplemented with bovine serum albumin (BSA, Sigma, USA). After washing, the cells were resuspended in PBS and fluorescent staining was measured using a flow cytometer (Beckton Dickinson, USA) and the results were analyzed with the Win MDI 2.8 software (Scripps Institute, La Jolla, CA, USA).

### Histotypic culture of ADSC, chondrocytes with dPNCG

Rat characterized ADSCs and chondrocytes were mixed with dPNCG in culture tubes, with culture medium (DMEM supplemented with 10% Fetal Bovine Serum (FBS), 1% L-glutamine) and incubated in 5% CO_2_ at 37 °C. The number of cells in the culture tubes ADSCs, chondrocytes (for 100% cells, 3×10^5^ cell/ml) as 100% with dPNCG (10 mg/mL) grown for 21 days.

### Quantification of GAG

The dye 1,9-dimethyl methylene binds to sulfated GAGs, the alterations in the absorption spectrum recorded allows fast detection of GAGs in solution. GAG content of the dPNCG and native nasal cartilage was quantified by spectrophotometric assays. Chondroitin 4 sulfate was used as a standard. The absorbance was read using a plate reader at 525 nm.

### Immunohistochemistry stain

Cultured ADSCs, chondrocytes and dPNCG, the 3D sample was sectioned into 5 μm sections and fixed on coated glass slides. Preheated 10 mM citrate buffer (pH 6.0) at 100 °C for 30 min for antigen retrieval was done. Immunostaining was done with a standard avidin-biotin-peroxidase complex detection kit (DakoCytomation, Glostrup, Denmark) according to the instructions for use. Primary antibodies were incubated for 60 minutes at room temperature, anti-aggrecan antibody, GTX54920, 1:100 dilutions, GeneTex, Inc. USA; anti-collagen II antibody, MAB1330, 1:100 dilutions, Merck, USA and secondary antibody Goat Anti-Rabbit IgG-Biotin, ab6720, 1:3000 dilutions, Abcam, Cambridge, MA, USAGoat Anti-Mouse IgG1-Biotin, ab98691, 1:2000 dilutions, Abcam, Cambridge, MA, USA, followed by the biotinylated secondary antibody for 1 h, and peroxidase-conjugated streptavidin for 30 min before washing with TBST three times for 5 min. The sections were covered with the DAB solution for 5 min to localize positive staining and counterstained with hematoxylin for 1 min, mounted and photographed and semi-quantified by ImageJ software (Wayne Rasband, National Institute of Health, USA).

### Statistical analysis

Statistical analyses were done using SPSS software, version 17 (IBM, Armonk, NY, USA). Besides, comparisons between groups were analyzed by Student's t-tests. Results were expressed as mean ± SD, P values of less than 0.001, 0.01 and 0.05 were considered statistically significant for different tests.

## Results

### Characterization of dPNCG

**Figure 1:**
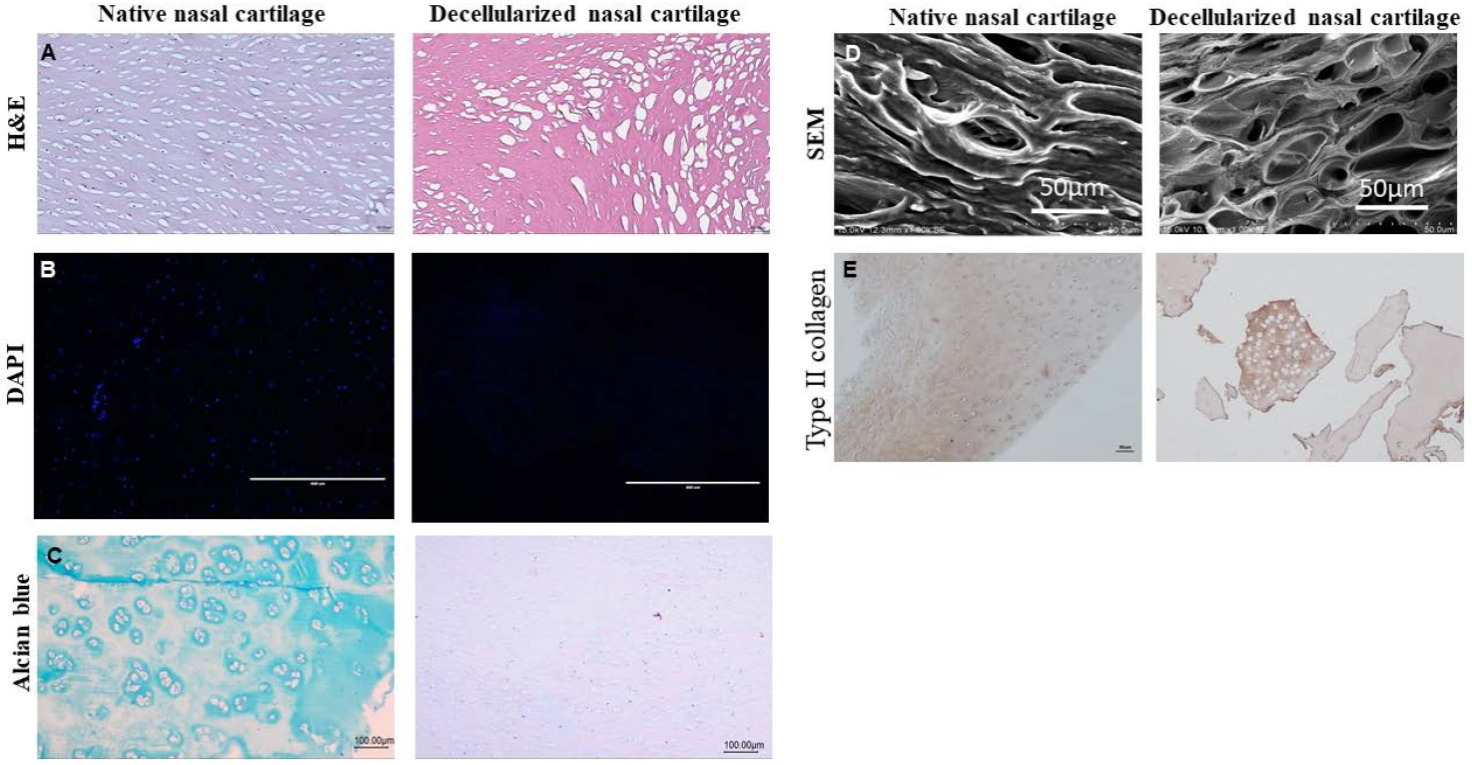
Characterization of dPNCG by (A) H&E staining of normal cartilage and dPNCG. (B) DAPI staining of normal cartilage and dPNCG. (C) Alcian blue staining of normal cartilage and dPNCG. (D) SEM of normal cartilage and dPNCG. (E) Type II collagen immunostaining of normal cartilage and dPNCG granules.

H&E staining depicted no cells and cell debris indicating complete decellularization of dPNCG relative to native nasal cartilage. In native nasal cartilage, the nucleus of the cell is stained blue-purple colour by hematoxylin and eosin stains the protein showing pink colour (Figure 1A). In DAPI staining, the native nasal cartilage showed blue fluorescent colour stained nucleus; however, dPNCG depicted no obvious nucleus which confirms complete decellularization (Figure 1B). Glycosaminoglycans (GAGs) were stained by alcian blue staining revealed in normal nasal cartilage depicted blue colour staining. However, in the dPNCG revealed no alcian blue indicating complete removal of GAG relative to native nasal cartilage (Figure 1C). SEM shows the lacunae with chondrocytes in native nasal cartilage; however, scCO_2_ decellularized dPNCG shows clear lacunae without chondrocytes (Figure 1D). In type II collagen immunostaining, the native nasal cartilage and dPNCG expressed type II collagen, demonstrating scCO_2_ did not alter the type II collagen content and confirms complete decellularization (Figure 1E).

**Figure 2:**
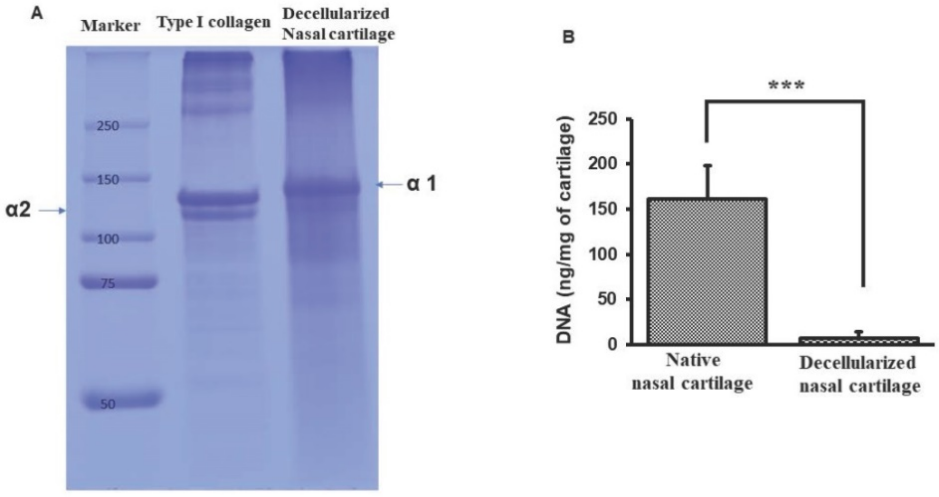
Characterization of dPNCG by (A) SDS-PAGE of collagen from dPNCG, Lane-1, molecular weight marker. Lane-2, type I collagen. Lane-3, dPNCG. (B) Quantification of DNA in normal cartilage and dPNCG. Results were expressed as mean ± SD, *** P < 0.001 were considered statistically significant for different tests (N=3).

SDS-PAGE lane 1, shows protein marker molecular weight ranging from 250-50 kilodaltons. Lane 2, type I collagen showed both αI and αII bands. Lane 3, dPNCG depicting only α1 band of type II collagen (Figure 2A). The permissible level of residual DNA content for a medical implant device is < 50 ng/mg. And in this study, the dPNCG DNA quantification revealed below 5 ng/mg and showed a significant (p<0.001) decrease in the DNA content relative to normal nasal cartilage (Figure 2B).

### Characterization of isolated rat ADSCs

**Figure 3:**
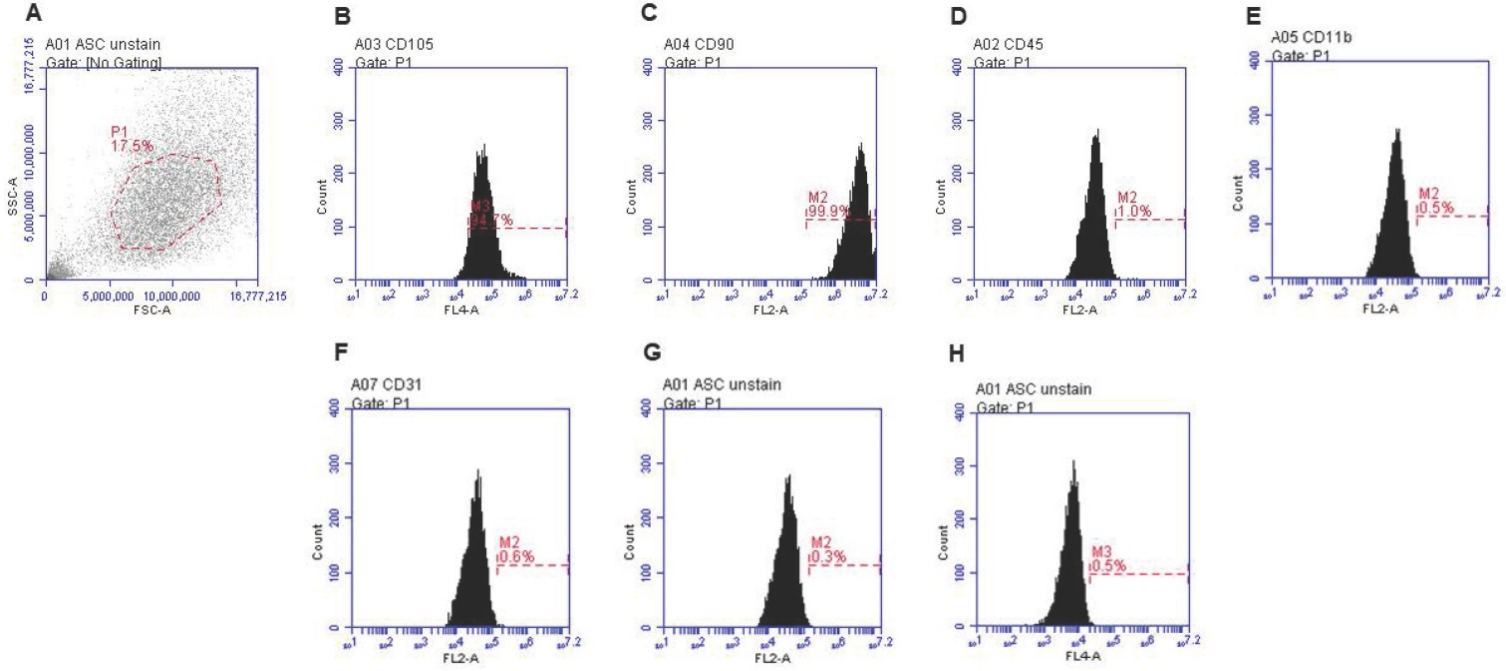
Isolation and characterization of rat ADSC. A-H Immunophenotype of rat ADSC. Immunophenotype of rat ADSCs analyzed by flow cytometry. Most cells expressed CD105, and CD90, but were CD45-negative, CD31-negative and CD11b-negative (N=3).

Rat ADSCs isolated revealed spindle-shaped morphology at passage 1. *In vitro* expansion of rat ADSCs depicted a fibroblast-like shape at passage 3. Using flow cytometry surface markers of rat ADSCs were evaluated at passage 4 for characterization and confirmation. As depicted in Figure 3A-H, the maximum number of the cells expressed CD105 (94.7%) and CD90 (99.9%), whereas negligible cells were positive for CD45, CD11b, and CD31. Unstained ADSC we showed in Figure 3G and H.

### Characterization of isolated rat chondrocytes

**Figure 4:**
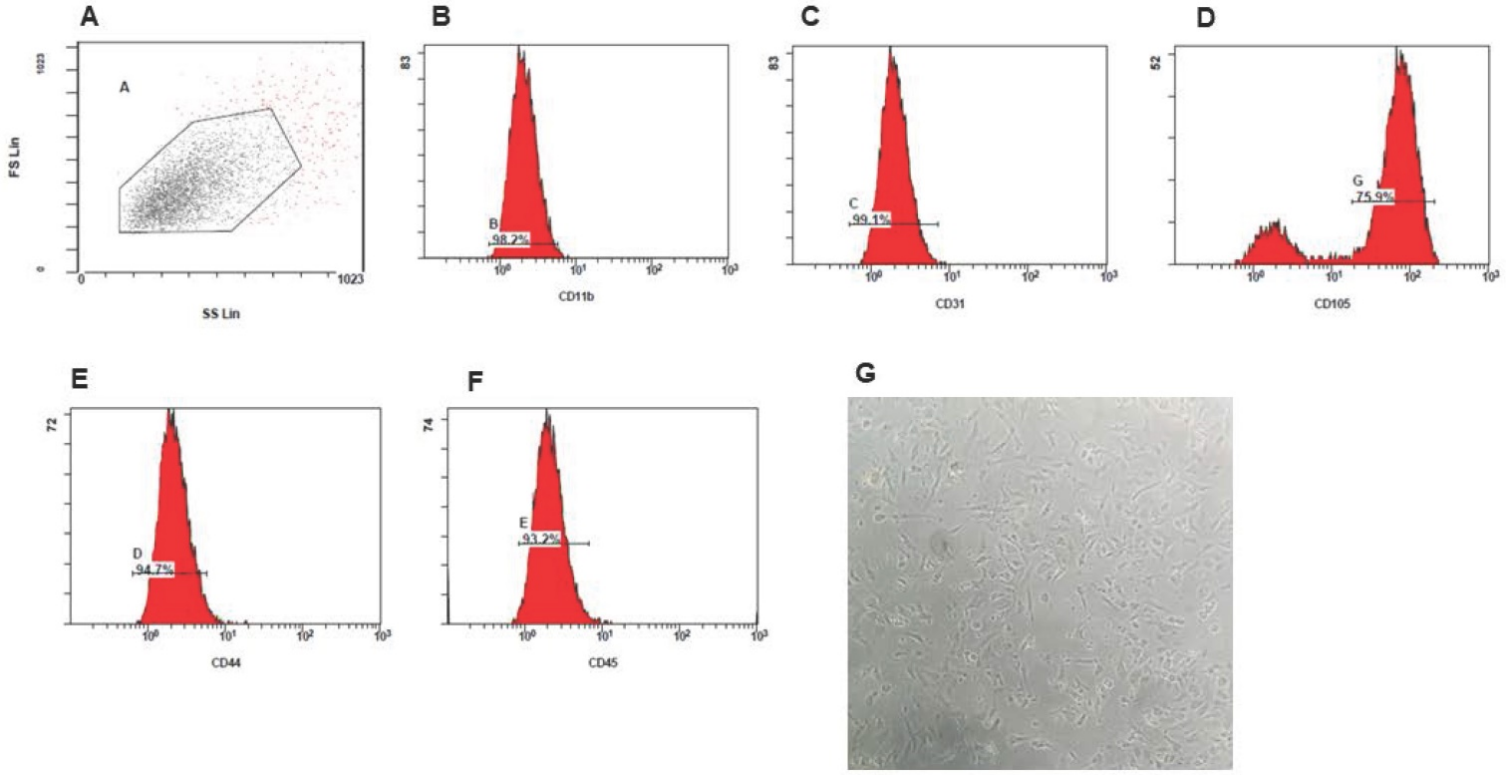
Isolation and characterization of rat chondrocytes. A-F Immunophenotype of rat chondrocytes. Immunophenotype of rat chondrocytes analyzed by flow cytometry. Most cells expressed CD11b, CD31, CD105, CD44, and CD45. G. Morphology of rat chondrocytes displayed spindle-shaped (N=3).

Immunophenotypic analysis of rat chondrocytes cultured at passage 1 showed spindle structure. *In vitro* expansion of rat chondrocytes depicted fibroblast-like shape at passage 3 (Figure 4G). Using flow cytometry surface markers of rat ADSCs were evaluated at passage 4 for characterization and confirmation. As depicted in Figure 4A-F, the most number of the cells expressed surface markers such as CD44 (94.7%), CD105 (75.9%), CD45 (93.2%), CD11b (98.2%) and CD31 (99.1%). These primary cultured chondrocytes did not possess the characteristics of ADSCs.

### Histotypic cultured 3D structure using SCCO2 decellularized dPNCG

**Figure 5:**
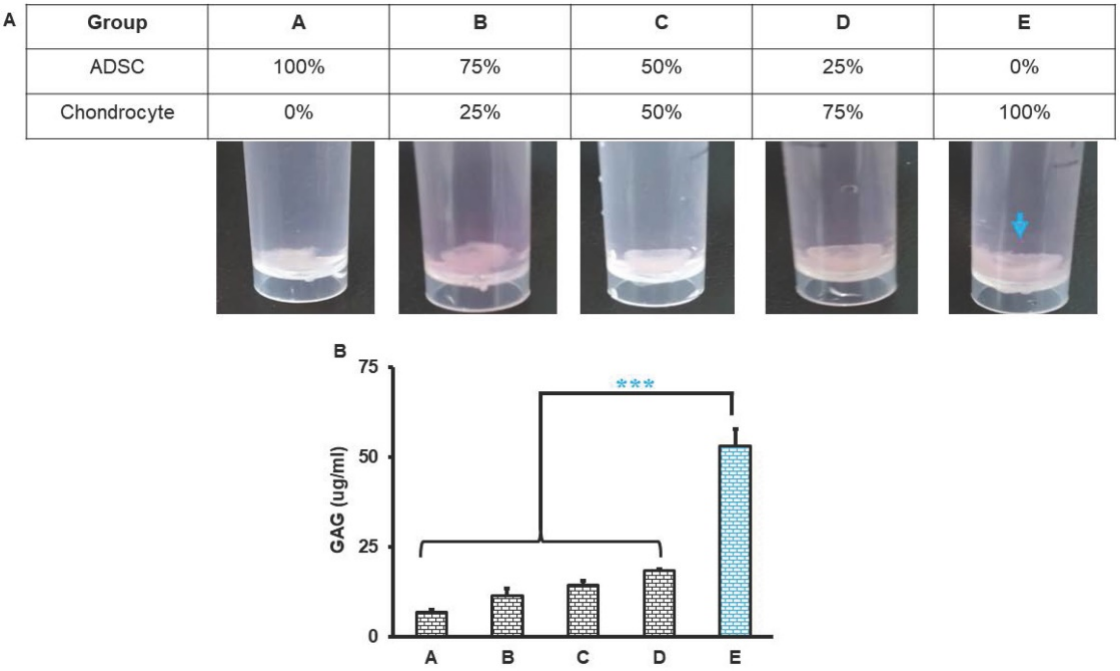
Histotypic culture of ADSC, chondrocytes with dPNCG. A. Grouping and representative photograph of rat ADSC and chondrocytes histotypic coculture with dPNCG. B. Quantification of GAG in the rat ADSC and chondrocytes histotypic coculture. Results were expressed as mean ± SD, *** P < 0.05 were considered statistically significant for different tests (N=3).

The triads of tissue engineering are cells, scaffolds and signals. We engineered histotypic 3D culture with combinations of different elements. Primary rat-derived ADSCs and chondrocytes culture reached 85-90% confluence at day 5 and passage 2 were used for histotypic 3D culture. The rat-derived ADSC and chondrocyte maintained their morphology during subcultures. Histotypic 3D culture was engineered with ADSCs, chondrocytes and dPNCG scaffold. After 21 days of culture obvious mass of tissue was observed in group D (25% ADSC and 75% chondrocytes) and E (0% ADSC and 100% chondrocytes), indicating the chondrocytes contributes to the formation of histotypic cultured 3D structure (Figure 5A). Quantification of the GAG revealed, a significant (P< 0.001) increase in the concentration of GAG in the group E (0% ADSC and 100% chondrocytes), indicating the chondrocytes secretes extracellular matrix and GAG that contributes to the formation of histotypic cultured 3D structure (Figure 5B).

### H & E and alcian blue staining of histotypic cultured 3D structure

**Figure 6:**
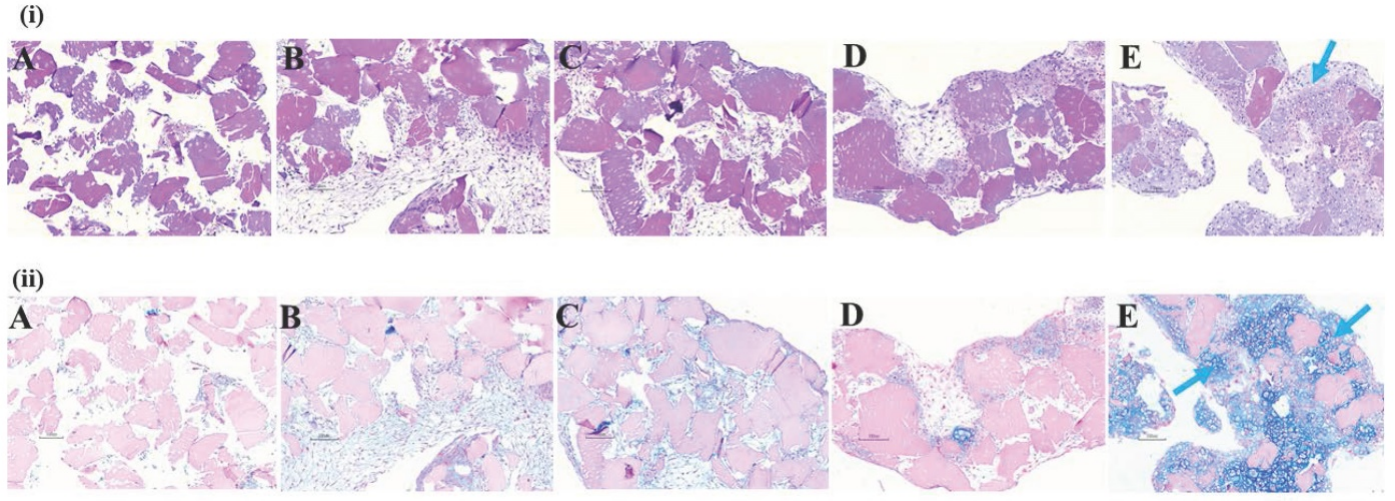
Representative photograph of rat ADSC and chondrocytes histotypic coculture with dPNCG. (i). Hematoxylin and eosin staining of products of histotypic coculture. (ii). Alcian blue staining of products of histotypic coculture. Blue arrows indicate secretion of extracellular matrix to form a solid mass of cartilage tissue with GAG secretion by chondrocytes.

H & E and alcian blue staining of histotypic cultured 3D structure revealed the cell growth, tissue mass formation and GAG formation in the 3D structure. H & E staining from group A to group C showed loose structure with spaces in between the dPNCG granules with cell scattered around the cartilage granules. However, in group D (25% ADSC and 75% chondrocytes) and E (0% ADSC and 100% chondrocytes) showed an intact mass of tissue, with cartilage granules bound to one another by extracellular matrix, to form a 3D structure (Figure 6). Alcian blue staining from group A to group D showed negative staining for GAG with scattered cartilage granules. However, group E (0% ADSC and 100% chondrocytes) depicted intense blue colour by alcian blue staining of the 3D histotypic structure formed with an excellent build of tissue, with cartilage granules as a single unit wrapped by extracellular matrix, indicating the chondrocytes contributes to the formation of histotypic cultured 3D structure (Figure 6).

### Phenotype evaluation of histotypic cultured 3D structure

**Figure 7:**
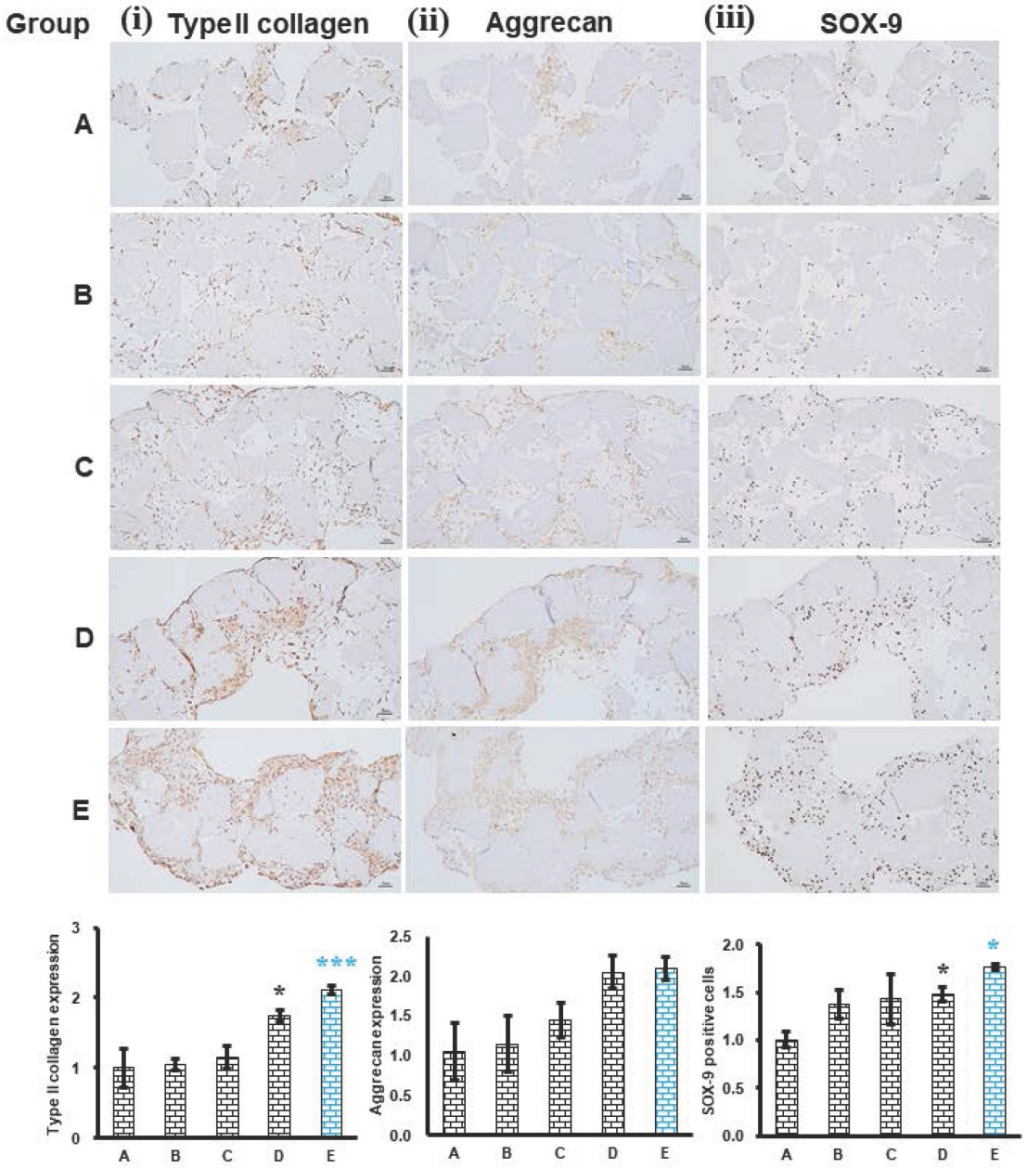
Histotypic culture of ADSC, chondrocytes with dPNCG. Phenotypic markers type II collagen (i), aggrecan (ii) and SOX-9 (iii) were examined by immunohistochemistry and semi quantification respectively. Results were expressed as mean ± SD, * P < 0.05, *** P < 0.001 were considered statistically significant for different tests (N=3).

The expression of phenotype chondrogenic markers, type II collagen, aggrecan and SOX-9 of histotypic cultured 3D structure was evaluated. The expression of type II collagen was significantly increased in group D (25% ADSC and 75% chondrocytes) (P<0.05) and group E (0% ADSC and 100% chondrocytes) (P<0.01) relative to group A (100% ADSC and 0% chondrocytes), indicating the chondrocyte in the histotypic culture is responsible for the secretion of type II collagen (Figure 7 (i)). The expression pattern of aggrecan was similar to that of type II collagen and SOX-9 which increased as the percentage of chondrocyte increased in group D (25% ADSC and 75% chondrocytes) and group E (0% ADSC and 100% chondrocytes) compared to group A (100% ADSC and 0% chondrocytes), demonstrating chondrocytes play a key role in the production of aggrecan which offers a hydrated gel structure that links type II collagen for the appropriate functioning of cartilage in the histotypic culture (Figure 7 (ii)). The expression of SOX-9 was significantly increased in group D (25% ADSC and 75% chondrocytes) (P<0.05) and group E (0% ADSC and 100% chondrocytes) (P<0.05) relative to group A (100% ADSC and 0% chondrocytes), the increase in SOX-9 expression indicates the proliferating chondrocytes were not hypertrophic chondrocytes, thus forming normal cartilage histotypic culture (Figure 7 (iii)). The expression of phenotype chondrogenic markers, type II collagen, aggrecan and SOX-9 in group E (0% ADSC and 100% chondrocytes) demonstrates the chondrogenesis in the dPNCG scaffold producing type II collagen and extracellular matrix which are bound together to form a 3D cartilage histotypic culture, which can potentially be used in the cartilage defect repair or cartilaginous augmentation, for example, fillers for rhinoplasty.

## Discussion

The quality of the life is greatly affected by nasal cartilage pathologies, a common form of facial abnormalities. The demand for cartilage quality and quantity is highly associated with donor site morbidity and possible surgical complications, including infection and resorption, which limits the ability to correct nasal cartilage pathologies. Nasal reconstruction involves the restoration of degraded or lost tissue. Cartilage tissue engineering offers solutions to the nasal defects that produce a filler of smaller defects, a scaffold to promote repair and a full implant as structural support. In the present study, we evaluated the role of scCO_2_ processed dPNCG cultured with ADSCs and chondrocytes for potential future rhinoplasty application. We found dPNCG cultured with chondrocytes led to the production of a 3D cartilage histotypic culture, which can be used as a full implant for structural support in rhinoplasty.

Cartilage decellularization is a challenging process, because of its dense structure with separate lacunae. Usually, cartilage decellularization is executed through the diffusion of detergents into the lacunae to lyse the chondrocytes. Subsequently washing out the remaining debris and nucleic acids. The cartilage was treated with 0.05% Trypsin/EDTA for one day followed by 3% SDS for two days and 3% Triton X-100 for another 2 days 13. The process comprises a combination of physical, chemical, and enzymatic steps 7, 13. SDS and Triton X-10 decellularization of cartilage resulted in only a 77% reduction in DNA content (262±42 ng/mg) compared to the non-treated cartilage sample. However, the main criteria for medical device, the DNA content in decellularized tissue have been defined to be less than 50 ng/mg in graft materials. Nevertheless, the dense compact nature of the reticular network of fibrous ECM in cartilage is a significant barrier for the detergents to penetrate. This is the major limitation of SDS and Triton X-100 decellularization of cartilage 13, 14. In another separate study, cartilage decellularization by SDS (2%) treatment for 4 or 8 hours resulted in complete decellularization. However, 60% of the DNA remained in the decellularized cartilage tissue 15. In the present study, the characterization of dPNCG was carried out to validate the complete decellularization, competent to be employed in cartilage histotypic culture for nasal cartilage defect reconstruction. dPNCG contains little or no genetic material as demonstrated by H&E and DAPI staining. The quantification of residual DNA showed a negligible amount in the decellularized cartilage. Decellularization by chemicals had been shown to contain high amounts of DNA fragments and damage to scaffold structure 5, 8.

The advantage of this new dPNCG production process uses scCO_2_ extraction technology for the production of cartilage graft which does not involve any chemicals. scCO_2_ uses mild critical coordinates, such as pressure at 7.38 MPa and temperature at 31°C, which can be easily accomplished and well‐suited for cartilage graft preparation. Supercritical CO_2_ at fluid state instantaneously displays gaseous viscosity and diffusion properties at temperature and pressure conditions above the critical point. The high transfer rate and high permeability of supercritical CO_2_, which can be changed continuously by varying the temperature and pressure. Therefore, supercritical CO_2_ easily and efficiently penetrates the cartilage and breaks, dissolves the chondrocytes that reside in the lacunae. Besides, the scCO_2_ produced dPNCG do not contain any chemical solvent residue, oil and lipids. The scCO_2_ process involves bactericidal and viral inactivation, which results in dPNCG with excellent biocompatibility. Also, the scCO_2_ process is natural, safe, non-toxic, non-corrosive, non-flammable, easily accessible, cost‐effective, destroys, and eliminates pathogens. There are no known disadvantages of the scCO_2_ technique regarding the production of decellularized collagen materials, including cartilage in literature 5, 8, 9, 10. Our previous related publication demonstrated the scCO_2_ process completely removed the cellular proteins, except collagen fibrils. However, in chemical detergent Trition X- 100 decellularization resulted in presence of cellular proteins that might cause adverse biocompatibility 9. Our other previous related publication demonstrated the scCO_2_ process drastically facilitates the enzymatic splicing of the telopeptides from collagen triple helical structure and thus helps in the complete removal of telopeptides from collagen scaffold. The major antigenic determinants are situated in the telopeptide region of collagen. Therefore, complete removal of the telopeptides by scCO_2_ process eliminates the adverse immune reaction with excellent biocompatibility 8. Therefore, our cartilage graft production is relatively superior to other existing cartilage grafts.

In the current study, H&E staining showed complete removal of chondrocytes inside the lacunae with intact cartilage scaffold. Analysis of the chemical content of dPNCG showed type II collagen, with no carbohydrate moiety or proteoglycans, indicating the efficiency of scCO_2_ technology that removed the cell debris from the cartilage scaffold. In the current study, scanning electron microscopy structural analysis of dPNCG developed by scCO_2_ technology depicted the native intact collagen scaffold with its clean lacunae. Clean lacunae might play a significant role in the efficacy of chondrocyte seeding, diffusion, and proliferation. On contrary, detergent SDS possesses a high affinity for proteins, denatures and eradicates ECM growth factors, and causes inadequate decellularization. Destruction of ECM components leads to the compromise in the mechanical properties of the cartilage graft. The risk of residual chemicals is another drawback with decellularization using SDS 13, 16-18.

ADSCs had been proven to possess chondrogenic properties and suggested they might retain the superior potential for long-term cartilage function than differentiated chondrocytes. SDS and Triton X-100 decellularized cartilage limited hADSCs cell proliferation and penetration inside the cartilage scaffold at 7 and 14 days. The dense compact reticular fibrous nature of the decellularized cartilage limited cell penetration. Therefore, this is the most substantial limitation of application in recellularization 13. Especially, *in vitro* chondrocytes progressively lose the capability to produce type II collagen over each passing generation. However, ADSCs retain and maintain the better chondrogenic potential for over 15 passages 19. ADSCs suppress the local immune response, which might play an important role in their regenerative capabilities. Therefore, ADSCs therapies benefits autoimmune cartilage disorders and effectively treat chondral lesions in osteoarthritis 19, 20. The tissue-engineered biomaterial scaffolds with ADSCs had been reported to enable the attachment, proliferation, and differentiation of embedded cells *in vivo* and *in vitro*. ADSCs cultured with several scaffolds in chondral defects with reparative hyaline cartilage that consisted of significant amounts of type II collagen, indicating good chondral repair, whereas without ADSCs showed fibrosis in the defect 20-22. SDS is the extensively used chemical in decellularization procedures that works by dissolving cell and nucleic membranes. However, adverse effects have been reported of SDS-treated scaffolds on recellularization 23, 24. The residues of the SDS are the main cause of the recellularization issue due to the matrix variations 25. The negative impact of SDS treatment on cell viability was observed not only restricted to the treated site but also in surrounding areas, demonstrating the release of a cytotoxic substance from the decellularized tissue. Cartilage appears to retain SDS to a higher extent. Therefore, the need for the replacement of SDS for cartilage decellularization is obviously needed 26.

Chondrocytes are regularly used in the nasal cartilage tissue engineering approach. Nasal chondrocytes sources from ovine 3, 27 rabbit, bovine 28, and human 29, 30 have been studied for nasal cartilage engineering. The sourcing of these cells is from animals, because of availability and cost in cartilage tissue engineering. Nowadays, human chondrocytes are most frequently investigated for nasal cartilage engineering, due to the availability of septal leftovers from rhinoplasty surgeries. Passages 3 of human nasal septal chondrocytes have the competence to produce neocartilage comprising GAG and type II collagen 30-32. Besides, auricular chondrocytes were also used for the investigation to explore the production of elastic or hyaline neocartilage. Instead, costal cartilage might be an abundant source of chondrocytes, because the harvesting might not cause morbidity or weakness in the cartilage structures of the nose. Cultured costal chondrocytes can produce neocartilage which is capable of remodelling *in vivo* to promote healing 30, 33.

In cartilage, huge multi-molecular aggregates of aggrecan were found, contained numerous monomers non-covalently linked to HA. Large mass aggrecan aggregates were formed by the contribution of GAG side chains. Aggrecan seems to be significant in facilitating chondrocyte-chondrocyte and chondrocyte-matrix interactions. Chondrocytes that express high concentrations of aggrecan and link protein were preserved within a matrix network and were able to survive in suspended culture 34. Cartilage decellularization by SDS (1%) treatment for 24 h and Triton X-100 (2%) treatment for 48 h retained the majority of the ECM components after a complete cell removal. The removal of chondrocytes during decellularization and the migration of seeded cells into the scaffolds during recellularization is challenging. The SDS used in the decellularization process resulted in the denaturation of protein structures, which may also destroy the protein function 35. In the present study, chondrocytes with dPNCG produced a tissue mass with GAG, extracellular matrix, aggrecan and type II collagen in the 3D histotypic culture, indicating the role of aggrecan in maintaining the extracellular matrix to form a 3D histotypic culture.

Chondrocytes residing in cartilage are accountable for the preservation of the specific extracellular matrix and its biomechanical properties. Chemical decellularized cartilage nasal graft seeded with chondrocytes resulted only by superficial infiltration of chondrocytes 36, indicating decellularized cartilage nasal graft is dense compact thus limiting the proliferation of seeded chondrocytes. Chondrocytes and extracellular matrix integrity depend on the expression of numerous matrix genes, including type II collagen, aggrecan and cartilage link protein, regulated by anabolic and catabolic transcription factors 37, 38. SOX-9 is an anabolic transcription factor, which belongs to the SOX family and plays an important role during cartilage development, expressed in chondrocytes. SOX-9 has been shown to contribute to chondrogenesis by activating cartilage-specific genes such as type II collagen and aggrecan 37, 39. SOX-9 might be involved in maintaining the chondrocyte phenotype in normal cartilage 37, 38. In the present study, chondrocytes with dPNCG produced a tissue mass with the extracellular matrix, aggrecan and type II collagen. The regulation of the type II collagen and aggrecan expression was modulated by SOX-9 in maintaining the chondrocytes and extracellular matrix integrity to form a 3D histotypic culture.

To conclude, the present investigation demonstrates that we had developed a non-toxic, biocompatible dPNCG by scCO_2_ technology. This dPNCG powder cultured with chondrocytes can produce a 3D histotypic functional cartilage tissue mass with expression of GAG, extracellular matrix, aggrecan and type II collagen. In which, dPNCG acted as a scaffold matrix for chondrocytes to produce type II collagen, aggrecan facilitates chondrocyte-chondrocyte and chondrocyte-matrix interactions. The SOX-9 acted in maintaining the chondrocytes and extracellular matrix integrity to form a 3D histotypic functional cartilage tissue, which can be extrapolated to repair/augment the cartilaginous defect for human rhinoplasty.
